# Depicting Developing Trend and Core Knowledge of Primary Open-Angle Glaucoma: A Bibliometric and Visualized Analysis

**DOI:** 10.3389/fmed.2022.922527

**Published:** 2022-07-05

**Authors:** Liting Zhao, Jinfei Li, Lemeng Feng, Cheng Zhang, Wulong Zhang, Chao Wang, Ye He, Dan Wen, Weitao Song

**Affiliations:** ^1^National Clinical Research Center for Geriatric Diseases, Xiangya Hospital of Central South University, Changsha, China; ^2^Xiangya School of Medicine, Central South University, Changsha, China; ^3^Eye Center of Xiangya Hospital, Central South University, Changsha, China; ^4^Hunan Key Laboratory of Ophthalmology, Changsha, China

**Keywords:** primary open angle glaucoma, intraocular pressure, bibliometrics analysis, citespace, VOSviewer

## Abstract

**Objective:**

The prevalence of glaucoma is rising due to an increasing aging population. Because of its insidious and irreversible nature, glaucoma has gradually become the focus of attention. We assessed primary open angle glaucoma, the most common type of glaucoma, to study its present status, global trend, and state of clinical research.

**Methods:**

Publications from 2000 to 2021 in Web of Science database were retrieved and analyzed by bibliometrics. VOSviewer and Citespace were used for analysis.

**Results:**

A total of 6,401 publications were included in this review, and we found that the number of publications increased from 139 in 2000 to 563 in 2021. American researchers have published the most papers and had the highest h-index and the most citations, while the *Journal of Glaucoma* has published the most papers on this topic. Some key researchers, contributing institutions, their partnerships, and scientific masterpieces were identified. The publications we reviewed fall into seven categories: publications on intraocular pressure, normal tension glaucoma, risk factors, the trabecular meshwork, optical coherence tomography, surgery, and mutation. Clear study hotspots were described, which began with epidemiology and transitioned to pathogenesis and diagnosis and then to treatment.

**Conclusion:**

Studies on primary open angle glaucoma extend well beyond ophthalmology to biochemistry molecular biology, general internal medicine, pharmacology, pharmacy, science technology, and other areas. Interest, research and publications on primary open angle glaucoma are on the rise.

## Introduction

Glaucoma affects nearly 80 million people and is the leading cause of irreversible blindness globally (1, 2). It is classified as primary glaucoma and secondary glaucoma based on the etiological mechanism. Primary glaucoma can be categorized into primary angle-closure glaucoma and primary open angle glaucoma (POAG) according to the anterior chamber angle morphology (3). The prevalence of the same type of glaucoma varies by region (4). The most common type of glaucoma is POAG—which is a complex genetic disease characterized by the loss of retinal ganglion cells and damage to the optic nerve, leading to progressive visual field loss (3, 5). The global prevalence of POAG among people aged 40–80 years was estimated to be 3.05% (1.69–5.27%) and 5.9 million people were blind due to this disease in 2020 (1). However, the severity of the problem may be greater than these figures indicate because epidemiological studies have indicated that approximately 50% of POAG cases are not diagnosed because glaucoma is an insidious disease and with population aging, the severity of the problem will be further aggravated (6). Even so, the global trend of POAG has not been well analyzed. Bibliometric analysis is a feasible strategy to qualitatively and quantitatively summarize and predict research trends by evaluating the research of main authors, journals, research institutes, and countries (7–9). In addition, bibliometric analysis has contributed to clinical decision-making and the development of guidelines (10–12). The purpose of this study is to analyze the current state and the trend of clinical research regarding POAG.

## Method

### Data Source

Although several databases can meet the needs of global-level analyses (13), we chose Science Citation Index Expanded (SCI-Expanded, 1999-present) of the WoS Core Collection (WoSCC) database for our evaluation. The WoSCC database covers more than 12,000 international scientific journals with great impact and quality and is the most commonly applied database for bibliometric analysis (14, 15)^1^. Apart from the general literature search, it also possesses an important function of citation index searching, which is helpful for assessing the academic performance of literature in a specific field (16).

### Search Strategy

**Figure 1:**
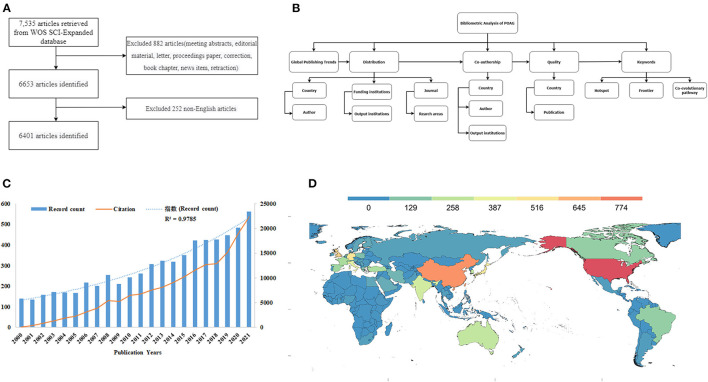
The methods of bibliometric analysis and the trends of POAG. **(A)** A flowchart representing retrieval strategies for POAG articles from the WOS SCI-Expanded database and the inclusion criteria for the study; **(B)** The whole flow chart of bibliometric analysis for POAG in this study; **(C)** Trends in the growth of publications and the number of cited articles worldwide from 2000 to 2021; **(D)** The distribution world map of POAG.

The topic was “primary open angle glaucoma,” and the “topic” field contained the title, abstract, author keywords, and keyword plus. We focused on publications between 2000 and 2021, and the search date was April 7, 2022. We selected “articleORreview” as the article type, excluded other language than English and retrieved and analyzed 532 reviews and 5,869 articles. All searches were performed in one day to avoid database update bias. The detailed data retrieval strategies and inclusion criteria for this study are summarized in Figure 1A.

### Data Extraction

We downloaded the data extraction information of all identified publications, including the title, author(s), year of publication, country of submission, affiliated institutions, journals, keywords, and abstracts. Two authors independently browsed and extracted data from eligible publications.

### Bibliometric Analysis

The basic feature of publications is the intrinsic function of WOS. The h-index is estimated for a scholar or scientist who has published h papers and each paper has been cited no less than h times by other studies (17). Therefore, the h-index determines the number of papers published by each researcher and all related citations to assess the productivity of the authors and the impact of the published research (18). Moreover, although H-index was initially developed to evaluate individual academic achievement, it could be extended to describe the publication output of a nation or region, an institution, or a journal (19).

### Visual Analysis

VOSviewer (Leiden University, Leiden, Netherlands) is a program for creating and visualizing bibliometric networks (20). In this study, VOSviewer was used for co-authoring, co-citation, and co-occurrence analyses. In the network map developed by VOSviewer, different nodes represent different elements, including authors, countries, institutions, and keywords, and the size of the node reflects the number or frequency of releases (21). The links between nodes represent associations, including co-authorship or co-occurrence, while the colors of nodes/lines reflect different clusters or years (22). The strength of the link is expressed in terms of total link strength (TLS). Co-authorship analysis illustrates the links between projects based on the number of co-authored papers. This is an effective tool for evaluating cooperation trends and identifying leading researchers, countries, and institutions (23). Co-occurrence analysis illustrates the relationship between keywords based on the number of publications where the keywords are found together (24). This analysis explores popular subjects and research directions. Therefore, it is an important indicator of the development of a particular research field. Similar to VOSviewer, CiteSpace is often used for literature analysis and visualization. It is used to capture keywords associated with strong citation bursts and explore keywords' time co-occurrence to predict research frontiers and explore keywords' co-evolutionary pathways (25). In this study, we used CiteSpace to make up for the gaps of VOSviewer. Microsoft Excel 2016 was used to predict the future trends of POAG publications. The equation of the prediction model was as follows: *f*_(x)_ = a^x^, in which x represented the publication year, and *f*_(x)_ represented the cumulative number of publications. In this way, we effectively captured the current status, emerging trends, and recent developments in the research of POAG. Figure 1B summarizes the entire process of bibliometric analysis.

## Result

Ophthalmic literature has been subjected to scientometrics in the past for glaucoma and specific journals to add insight to the evolving trends (26, 27). However, glaucoma is a general term which contains a large number of subspecies and the POAG is the most common type of glaucoma. The pathogenesis and epidemiology of different types of glaucoma are different, thus it is necessary to classify and discuss them separately in bibliometric analysis. Our research is mainly focused on POAG and is the first time to analysis the research of POAG specifically.

### Publication Output and Development Trend

The WOS database contains a total of 6,401 publications (532 reviews and 5,869 articles) related to POAG between 2000 and 2021. The distribution of articles was analyzed according to the year of publication (Supplementary Table 1). The report showed that from 2000 to 2021, the total number of publications increased from 139 to 563. The number of papers published in 2021 was the highest (563, 8.796%). The annual increase in publication and citations is shown in Figure 1C, which indicates that citation changes are roughly synchronized with the number of publications.

### The Distribution and Co-authorship Analysis of Countries, Authors, Funding Institutions, and Output Institutions

#### Distribution

##### Country

The countries that made the greatest contributions are presented in Supplementary Table 2. A distribution world map of POAG research is shown in Figure 1D. The US had the largest number of publications (1,898), with a centrality of 31%.

##### Author

The top 10 authors with the highest number of publications are listed in Table 1. Over the past 22 years, these authors have published a total of 874 publications, accounting for 13.65% of the global total publications. It is important to note that we included all the authors in our analysis regardless of their relative contributions (first author, corresponding author, or co-author).

**Table 1:** The top 10 authors in the study of POAG.

**Rank**	**Author**	**Country**	**Affiliate**	**Publications**	**TLS**
1	Weinreb RN	USA	University of California San Diego	127	280
2	Aung T	SINGAPORE	Singapore National Eye Center	127	258
3	Pasquale LR	USA	Icahn School of Medicine at Mount Sinai	104	122
4	Wiggs JL	USA	Harvard Medical School	95	139
5	Mackey DA	Australia	University of Western Australia	75	173
6	Ritch R	USA	New York Eye & Ear Infirmary of Mount Sinai	73	65
7	Allingham RR	USA	Duke University	70	111
8	Craig JE	Australia	Flinders University South Australia	70	192
9	Park KH	Korea	Seoul National University College of Medicine	69	155
10	Wang NL	China	Capital Medical University	64	64

*TLS, Total link strength*.

##### Funding Institutions

A total of 3,109 institutions funded the publication of POAG related works. Table 2 lists the top 10 funding institutions from 2000 to 2021. Seven of the top 10 are from the US, with one each from Japan, China and Europe.

**Table 2:** The top 10 funding institutions and output institutions in the study of POAG.

**Rank**	**Funding institutions**	**Country/Region**	**Publications**	**Output institutions**	**Country**	**Publications**	**TLS**
1	United States Department of Health Human Services	USA	939	University of California System	USA	324	486
2	National Institutes of Health NIH USA	USA	925	University of London	UK	267	359
3	Nih National Eye Institute Nei	USA	782	University College London	UK	223	347
4	Research to Prevent Blindness Rpb	USA	367	Harvard University	USA	212	317
5	National Natural Science Foundation of China Nsfc	China	248	League of European Research Universities Leru	Europe	206	528
6	Ministry of Education Culture Sports Science and Technology Japan Mext	Japan	153	National University of Singapore	Singapore	202	523
7	European Commission	Europe	125	Singapore National Eye Center	Singapore	194	550
8	Pfizer	USA	115	Duke University	USA	191	542
9	Abbvie	USA	107	Moorfields Eye Hospital Nhs Foundation Trust	Germany	183	116
10	Allergan	USA	103	Massachusetts Eye Ear Infirmary	USA	168	110

*TLS, Total link strength*.

##### Output Institutions

The top 10 output institutions are also shown in Table 2. A total of 4,306 institutions have participated in POAG research. Four of the top 10 institutions are from the US, while the other top output institutions are from the UK, Singapore, and Germany.

#### Co-authorship Analysis

We used VOSviewer to map the network, visually showing the links between countries, authors, and institutions that have contributed to POAG research.

##### Country

**Figure 2:**
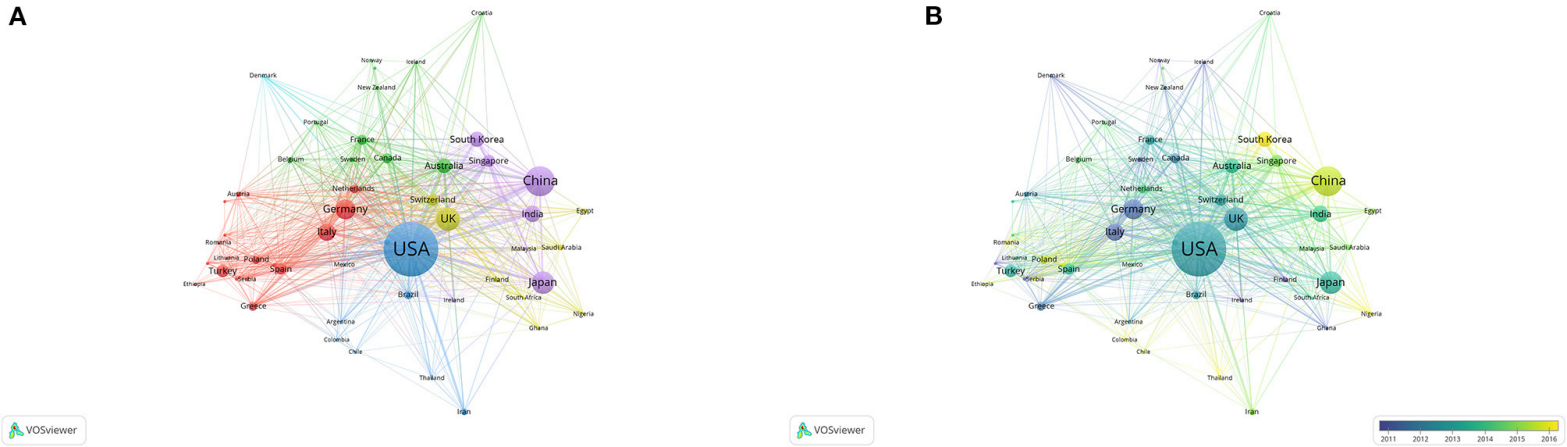
Cooperation map of countries in the studies of POAG. **(A)** Mapping of the co-authorship analysis amongst 69 identified countries. Each node represents an individual country, and the node size is proportional to the number of publications. Line thickness between nodes indicates link strength of a collaboration relationship (weighted by a quantitative evaluation indicator of TLS). **(B)** Country overlay. The color of each node represents the average year of POAG publications in a country. Blue represents the earlier published countries, while yellow represents the recently published countries.

A total of 69 countries with at least ten publications were identified. As shown in Figure 2A, the top five countries for TLS are as follows: the US (TLS = 1,399 times), the UK (TLS = 694 times), China (TLS = 448 times), Germany (TLS = 441 times), and Singapore (TLS = 416 times). The US has conducted extensive research on POAG and cooperated closely with other countries around the world in this research field to jointly promote the development of POAG research. The superimposed visualization map (Figure 2B) of co-authorship analysis shows that China, South Korea, Poland and other countries have also made some progress in the research field of POAG in recent years.

##### Author

**Figure 3:**
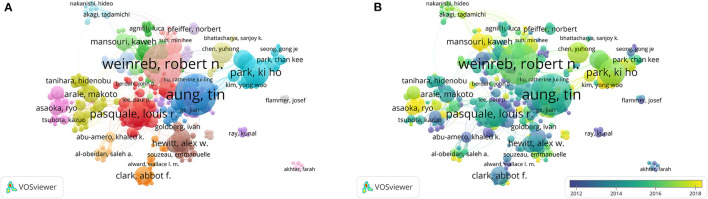
Cooperation map of authors in the studies of POAG. **(A)** Author network; **(B)** Author overlay. Explanatory figure legend is the same as for Figure 2.

Figure 3A shows the degree of collaboration among authors, with a total of 803 authors who have at least 10 publications. The top five authors with the highest TLS were Weinreb RN (TLS = 280), Aung T (TLS = 259), Craig JE (TLS = 192), Hewitt AW (TLS = 188), and Mackey DA (TLS = 173). The superimposed visualization map of co-authorship analysis was also performed in Figure 3B.

##### Output Institution

**Figure 4:**
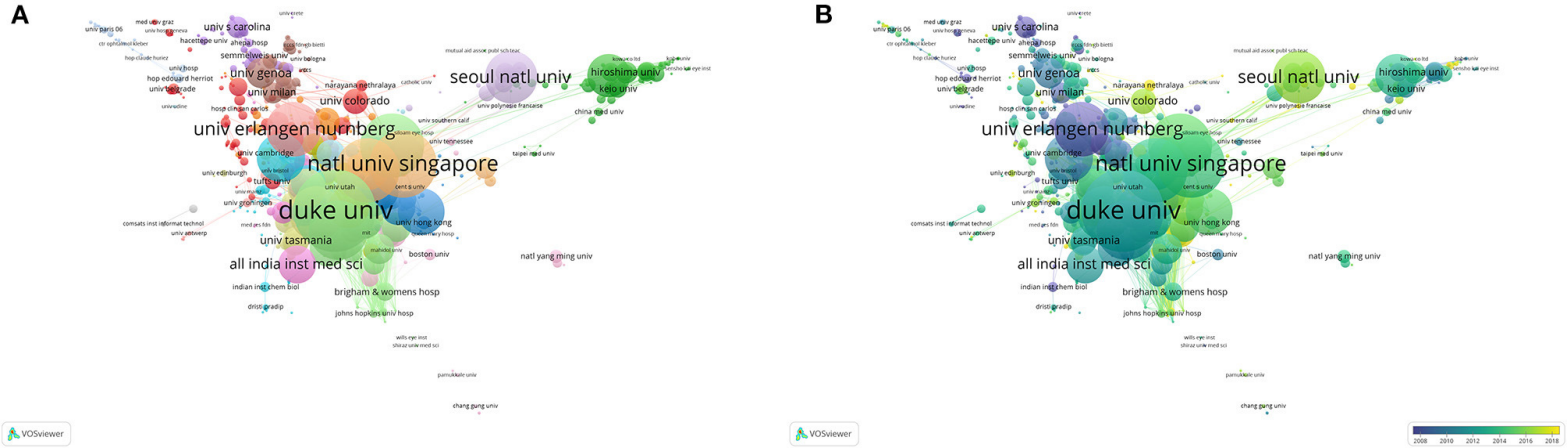
Cooperation map of output institution in the studies of POAG. **(A)** Institution network; **(B)** Institution overlay. Explanatory figure legend is the same as that of Figure 2.

As shown in Figure 4A, Duke University (542 TLS); the National University of Singapore (523 TLS); the Singapore National Eye Center (500 TLS); the University of California, San Diego (486 TLS); and the University of Melbourne (378 TLS) were the top five universities for TLS. According to the average output time, the superimposed visual map of co-authorship analysis was performed, as shown in Figure 4B.

### Journal Distribution and Research Areas

#### Journal Distribution

A total of 206 academic journals have published articles on POAG. Table 3 lists the top 10 most published journals and their impact factors.

**Table 3:** The top 10 journals and research areas in the study of POAG.

**Rank**	**Journal**	**Country**	**Publications**	**IF(2020)**	**Research areas**	**Publications**
1	Journal of glaucoma	USA	644	2.503	Ophthalmology	4601
2	Investigative ophthalmology visual science	USA	483	4.799	Biochemistry molecular biology	399
3	Ophthalmology	Netherlands	281	12.079	General internal medicine	364
4	American journal of ophthalmology	Netherlands	237	5.258	Science technology other topics	331
5	British journal of ophthalmology	UK	227	4.638	Pharmacology pharmacy	290
6	Molecular vision	USA	205	2.367	Research experimental medicine	265
7	Plos one	USA	201	3.240	Genetics heredity	238
8	Graefes archive for clinical and experimental ophthalmology	Germany	178	3.117	Neurosciences neurology	131
9	European journal of ophthalmology	Italy	168	2.597	Surgery	83
10	Eye	UK	155	3.775	Cell biology	79

*IF, Impact factor*.

#### Research Areas

The top 10 research areas among the major journals are also shown in Table 3.

### Analysis of the Quality of the Publications

The total number of citations and h-index reflect the quality of publications and the academic impact of one country (28). In addition to analyzing the number of publications in different countries around the world, we also analyzed the quality of publications in Table 4.

**Table 4:** The top five countries with the largest citation and highest H-index.

**Rank**	**Citation frequency**	**Average citation frequency**.	**H-index**
1	USA (81339)	Iceland (51.27)	USA (116)
2	UK (20577)	Singapore (40.62)	Germany (66)
3	Germany (16392)	Portugal (36.78)	UK (64)
4	China (13397)	USA (35.19)	China (54)
5	Japan (13064)	France (34.35)	Italy (53)

### The Top 10 Most Cited Publications

The average number of citations per paper on POAG was 26.55. Table 5 shows the 10 most cited articles about POAG.

**Table 5:** The top 10 most cited publications in the study of POAG.

**Rank**	**Title**	**TC**	**Author**	**Source Title**	**Published**
1	The ocular hypertension treatment study - a randomized trial determines that topical ocular hypotensive medication delays or prevents the onset of primary open-angle glaucoma	2,458	Kass MA, HeuerDK, Higginbotham EJ et al.	Archives of ophthalmology	Jun 2002
2	Global prevalence of glaucoma and projections of glaucoma burden through 2040 a systematic review and meta-analysis	2,325	Tham YC, Li X, Wong TY et al.	Ophthalmology	Nov 2014
3	The Ocular Hypertension Treatment Study - Baseline factors that predict the onset of primary open-angle glaucoma	1,791	Gordon MO, Beiser JA, Brandt JD et al.	Archives of ophthalmology	Jun 2002
4	The pathophysiology and treatment of glaucoma a review	1,404	Weinreb Robert N, Aung T, Medeiros FA et al.	Jama-journal of the american medical association	May 2014
5	Development of the 25-item national eye institute visual function questionnaire	1,371	Mangione CM, Lee PP, Gutierrez PR et al.	Archives of ophthalmology	Jul 2001
6	The ubiquitin kinase PINK1 recruits autophagy receptors to induce mitophagy	1,292	Lazarou M, Sliter DA, Kane LA et al.	Nature	Aug 2015
7	Primary open-angle glaucoma	1,238	Weinreb RN, Khaw PT	Lancet	May 2004
8	The impact of ocular blood flow in glaucoma	1,127	Flammer J, Orgul S, Costa VP et al.	Progress in retinal and eye research	JUL 2002
9	Mutations of optineurin in amyotrophic lateral sclerosis	861	Maruyama H, Morino H, Ito H et al.	Nature	May 2010
10	Adult-onset primary open-angle glaucoma caused by mutations in optineurin	788	Rezaie T, Child A, Hitchings R et al.	Science	Feb 2002

*TC, Total citation*.

### Keywords Analysis

#### Research Hotspot

**Figure 5:**
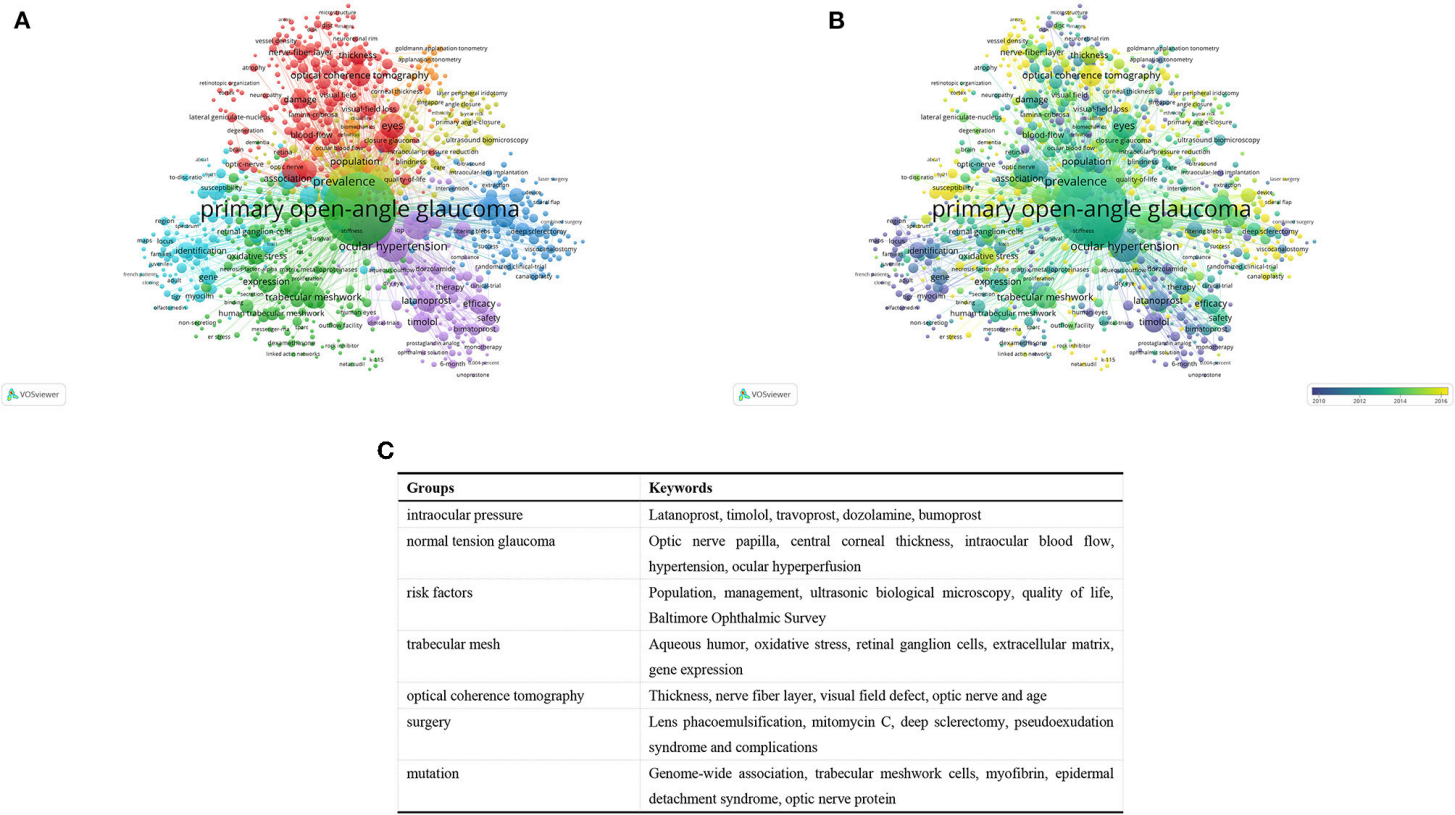
Co-occurrence analysis on POAG research. **(A)** Network visualization map of keyword co-occurrence analysis using VOS viewer. All keywords are labeled. The size of the node reflects the occurrence frequency of a certain keyword. The larger the size of the node is, the more frequently the keyword co-occurs. VOS viewer marks keywords with different colors, and the color of the nodes and labels indicates the cluster in which they belong to. Closely related keywords are grouped into one cluster with the same color. The higher the quantity of co-occurrences of two keywords, the closer will they be located in the network; **(B)** Overlay visualization map of keyword co-occurrence analysis. The meanings of the node and label in this map are the same as in Table 6. However, the color of each node in this map indicates the average year of the keyword in the article according to the color gradient in the lower right.

We identified and analyzed keywords that have been used more than 10 times in publications through VOSviewer. As shown in Figure 5A, 1,000 keywords can be grouped into approximately seven research clusters by color. Figure 5B shows an overlay visualization of co-occurrence analysis, where items are colored according to the average time in which the keywords appear. The most common keywords in each group are listed in Figure 5C.

#### Research Frontier

Burst keywords are frequently cited words over a period, indicating a sharp increase in the frequency of keywords, which can last for years (29). CiteSpace is used to detect burst keywords that are considered indicators of cutting-edge topics of research over time. As can be seen from the keywords listed in Table 6, “localization” showed the strongest outbreak intensity, followed by “timolol” and “locus,” etc.

**Table 6:** The top keywords with the strongest citation bursts.

**Keywords**	**Strength**	**Begin**	**End**	**2000-2020**
Localization	22.46	2000	2009	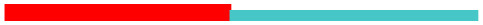
Timolol	21.28	2000	2006	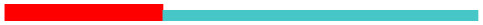
Region	15.61	2000	2009	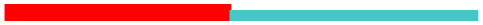
*Tiger* gene	14.44	2000	2004	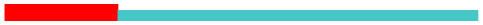
Baltimore eye survey	13.36	2000	2006	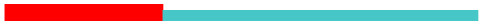
Mutation	12.36	2000	2007	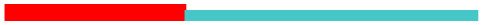
Prostaglandin analogs	10.83	2000	2006	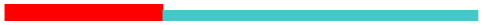
Locus	17.81	2002	2010	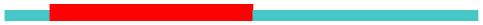
Elevated intraocular pressure	16.19	2002	2012	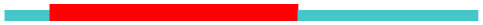
Fixed combination	12.72	2005	2010	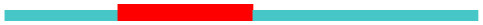
Genome wide scan	10.75	2005	2011	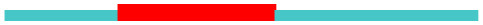
Filtering surgery	11.33	2006	2011	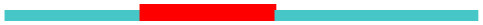
Optical coherence tomography	20.9	2016	2018	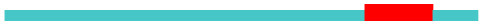
Vessel density	14.89	2017	2021	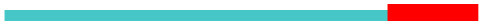
Coherence tomography angiography	14.99	2018	2021	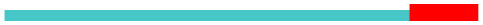

#### Co-evolutionary Pathway

**Figure 6:**
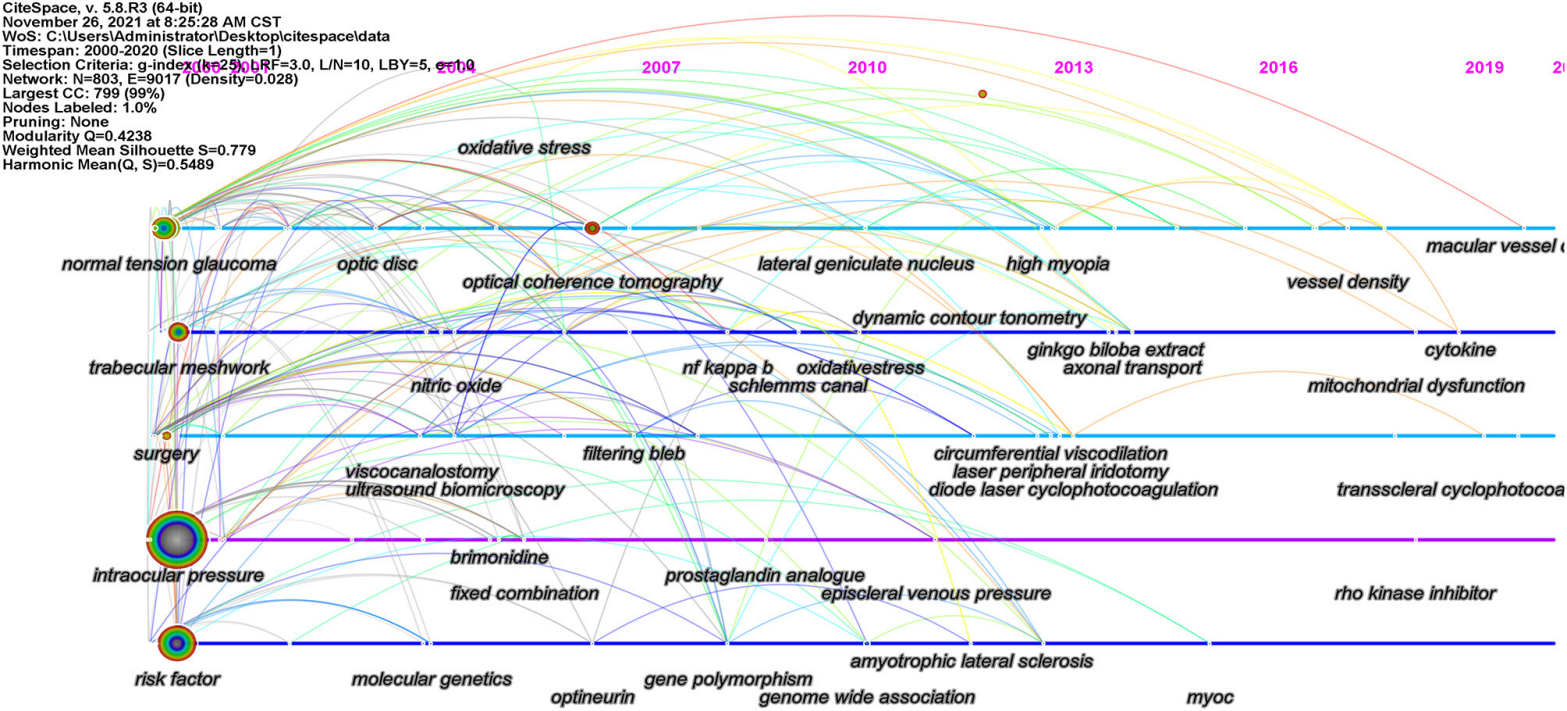
Evolutionary pathway in the study of POAG: The position of each node represents when it first appeared, and the lines between nodes represent relationships between keywords. The node colors represent different years, from cold to warm means period from 2000 to 2021. Bluish purple indicates the previous keyword, and red indicates the latest keyword. Longer colored segments indicate a larger reference time span. The flow of knowledge between clusters from cool to warm colors can also be observed over time.

Figure 6 shows the change in the keywords over time. Citespace is used to make co-evolutionary pathway and some significant keywords such as “normal tension glaucoma” (NTG), “trabecular mesh,” “surgery,” “intraocular pressure,” “risk factors” and “optical coherence tomography” are determined.

## Discussion

### Analysis of Research Results

In this analysis, 6,401 POAG related publications were found on WOS from 2000 to 2021. We observed that from 2000 to 2021, the number of POAG publications showed a wavy increase within 3–5 years. The search time for our survey was April 2022, so we believe all the publications in 2021 were included. During this 22 year period, the US, China, the UK, Germany, and Japan became the top five countries with the highest number of POAG related publications. This is roughly consistent with the distribution of the funding institutions. Singapore is not among the top 10 countries with publications, but two of the top 10 output institutions are in Singapore. In terms of global publication quality (specifically citation frequency and h-index), the US, the UK, and Germany are the top three countries; however, the average citation frequency of Iceland (51.27) is much higher than that of other countries. In addition, some other countries, such as Singapore, Portugal, USA, and France, also have good publication quality. The Journal of Glaucoma has the highest number of publications associated with POAG (644), but the journal with the highest impact factor is Ophthalmology (IF2020 = 12.079), the only one among the top 10 journals with an IF score > 10. The analysis of research hotspots showed that research fields of POAG go far beyond ophthalmology and that there will be some progress in biochemistry, internal medicine, pharmacology, research experimental medicine, genetics, neuroscience, cell biology, and other areas, as shown in Table 3.

The top 10 authors with the most publications were from the US, Singapore, and Australia. Dr. Weinreb RN and Dr. Aung T have published the same 127 articles over the past 22 years, making themself the most prolific author of the period. The most cited article of Dr. Aung T is “Global Prevalence of Glaucoma and Projections of Glaucoma Burden through 2040: A Systematic Review and Meta-analysis,” which has been cited up to 2,325 times and is the second most cited article among studies on POAG. His review suggests that the number of patients with glaucoma worldwide will increase to 111.8 million by 2040, with a higher proportion in Asia and Africa. These have important implications for glaucoma screening and treatment and the design of relevant public health strategies (2). Dr. Weinreb RN's most cited article is “The Pathophysiology and Treatment of Glaucoma: A Review,” which ranks fourth in terms of citations in this search area. This review further discussed the pathophysiological mechanism and treatment of POAG (3). Prof. Pasquale LR and Prof. Wiggs JL's conducted research on central corneal thickness in the US (30). The most widely cited publication in this field is “The Ocular Hypertension Treatment Study: A Randomized Trial Determines that Topical Ocular Hypotensive Medication Delays or Prevents the Onset of Primary Open-Angle Glaucoma,” which was published by Prof. Kass MA in 2002 with 2,458 citations in WOS. This paper suggests that topical ocular antihypertensive agents can effectively delay or prevent POAG in people with elevated intraocular pressure (IOP). While this does not mean that all patients with elevated IOP should be treated with medications, clinicians should consider initiating treatment for patients with elevated IOP who are at moderate or high risk of POAG (31).

The co-authorship map was constructed to visualize authors, countries and regions, and institutes that published at least 10 publications between 2000 and 2021. Not surprisingly, the top five authors who collaborated most closely were grouped among the top 10 authors who published the most. The top five most collaborative countries and territories (except for Singapore) and the top five institutions (except for Australia's University of Melbourne) were also in the top 10. We used co-occurrence cluster analysis to produce the network graph of co-occurrence relationship by analyzing the keywords found in the studies on POAG. A total of seven potential research directions were identified. Nodules of different colors (ranging from blue to red) showed considerable density in all seven clusters, suggesting a balanced pattern of development in all seven directions. In addition, research hotspots in each direction are changing, indicating that research is diversifying. The frequency of global cooperation has become closer, reflecting the growing trend of international and global cooperation among researchers who have interest in POAG.

### Research Trend Change Analysis

#### Overview of Research Trends Change

In keywords co-occurrence analysis, blue coded keywords, including “timolol,” “mutation,” “myofibrin,” “bromonidine,” “dozolamine,” “olfaction,” and “adjuvant therapy,” appeared earlier. After 2010, as more detailed studies were carried out, keywords such as “risk factors,” “normal intraocular pressure glaucoma,” “optic nerve fibers,” and “oxidative stress” began to appear. In the burst keywords analysis of Table 6, drugs such as “timolol” and “prostaglandin analogs” and keywords related to gene research, such as “localization,” “region,” “*TIGR* gene,” and “mutation,” appeared earlier. In addition, “scanning laser polarimetry” and “Baltimore Eye Survey” also emerged early. From 2000 to 2021, other keywords such as “locus,” “elevated intraocular pressure,” “fixed combination,” “genome-wide scan,” and “filtering surgery” broke out successively. Coherence tomography angiography has been developed based on OCT and vessel density in recent years. The keywords “vessel density” and “coherence tomography angiography” showed persistence after explosion in recent years, indicating that this field is the next research frontier. To further verify the research trend, co-evolutionary pathway is made: keywords such as “normal tension glaucoma” (NTG), “trabecular mesh,” “surgery,” “intraocular pressure,” and “risk factors” appeared frequently and early, which had a broad relationship with other clusters and represented the main research content and direction in the studies on POAG. OCT is an emerging hotspot for studies on POAG due to its late appearance and extensive connections with other clusters.

#### Diversity of Glaucoma Etiology

Because of the choice of database, our research focused only on publications from the 21st century, but the research content of the last century is the foundation. In the 1990s, James et al. (32) conducted detailed ophthalmic screening of residents of 16 clusters in East Baltimore to study the association of POAG with risk factors such as IOP, systemic hypertension, perfusion pressure, ethnic differences, family history, age, and optic disc structure. These findings are gradually being confirmed by studies in other communities or countries. In addition, other related risk factors have been identified in recent years. The Ocular Hypertension Treatment Study showed that the thinner the central cornea is, the greater the risk of developing POAG (33). In the Bayesian meta-regression model, men were more likely to develop POAG than women, and urban residents were more likely to develop POAG than rural residents (2).

In the 1950s, elevated IOP was thought to be a determinant factor for glaucoma. In patients with POAG, the increase in IOP is mainly due to increased resistance to outflow of aqueous humor through the trabecular meshwork (32). With the discovery of NTG and physiologic high IOP in recent years, we have realized that the increase in IOP alone cannot explain the whole pathogenesis of glaucoma development (34, 35). Berdahl et al. (36) put forward in 2008 that in patients with POAG and NTG, displacement of the ethmoid plate is mainly due to an increased pressure gradient of the ethmoid plate caused by a decrease in cerebrospinal fluid (CSF) pressure. Compression, deformation, and remodeling of the ethmoid plate lead to mechanical axon damage and disruption of axon transport, which in turn leads to damage to retinal ganglion cells (4). Therefore, the concept of transethmoid pressure difference was proposed to describe the difference between the preethmoid pressure (i.e., IOP) and the retroethmoid pressure (i.e., CSF pressure) (37). Studies have shown that in addition to lowering IOP, CSF pressure can be regulated through systemic therapy to rebalance the pressure gradient across the ethmoid plate in patients with NTG (38). Furthermore, studies have shown that high IOP can also change the environment of other retinal neurons and cells in the central visual pathway by causing microcirculation disorders, immune changes, excitatory toxicity, and oxidative stress or by increasing their sensitivity to injury (39). Genetic factors have long been implicated in the pathophysiology of POAG. *MYOC/TIGR* mutations are associated with some forms of adolescent open-angle glaucoma (40). With the development of genome-wide association analysis in recent years, several genes associated with POAG have been highlighted in previous reviews. The most mature genes include *CAV1, TMCO1, CDKN2B-AS1, Six6, ABCA1, GMDS, AFAP1, Gas7, TGFBR3, TXNRD2, ATXN2*, and *FOXC1*, and many of the associations in these genes have been repeated in multiple studies and in different ethnic populations (41). The mechanism by which these genes lead to POAG is unclear, but they may interact with transforming growth factor beta, a molecule that regulates cell growth and survival throughout the body to cause trabecular fibrosis and prevent aqueous outflow, which in turn leads to IOP and death of retinal ganglion cells (42).

#### Earlier Diagnosis of Glaucoma is Associated with Better Prognosis

Patients do not show any symptoms until the glaucoma progresses, leading to extensive nerve damage. When symptoms do occur, the disease causes irreversible vision loss and reduced visual field, so early diagnosis and intervention are crucial to slow the progression of the disease. Although IOP measurement alone is no longer the sole basis for the diagnosis of POAG, IOP remains a consistent risk factor for glaucoma. With the death of retinal ganglion cells and loss of optic nerve fibers in glaucoma, the appearance of the optic nerve head and the retinal nerve fiber layer changes characteristically (43). Examination of the optic nerve head with an ophthalmoscope can reveal signs of neuronal loss, but its appearance in healthy people is highly variable, making it challenging to identify early damage (44). OCT, which has been developing rapidly in recent years, is a hot research keyword lately. It can provide more objective and quantitative information about the loss of optic nerve fibers (retinal ganglion cell axons) (45). However, by the time OCT will show some morphological changes, the retina would have been permanently damaged. Therefore, we need technologies that can be used for early diagnosis and effective treatment. Doppler optical coherence tomography, also known as OCT angiography, is a technology that combines the Doppler effect principle with OCT. It can observe the capillary morphology and blood flow in the optic disc area of patients with glaucoma, which can be used as a sensitive index for early diagnosis of POAG (46).

#### The Only Effective Treatment to Slow Down the Progress of POAG is to Reduce IOP with Eye Drops, Laser or Surgery

Most patients with glaucoma prefer prostaglandins, as they reduce IOP by increasing aqueous drainage through the uveal sclera pathway (47). Other second-line drugs, such as β-adrenergic blockers, α-adrenergic agonists, and carbonic anhydrase inhibitors, are not as effective as prostaglandin analogs in reducing IOP and may have some side effects (48). When medication does not achieve sufficient IOP reduction with acceptable adverse reactions, laser or surgical procedures are recommended. In recent years, the continuous improvement of surgical methods for glaucoma, the advancement in technology, the research of postoperative antiscar formation drugs, and the invention and implication of new implants have brought the surgical treatment of POAG to a new field. While a substantial reduction in IOP can be achieved in most patients, the effect diminishes over time, with an annual failure rate of about 10% (49). Some patients still have impaired visual function after normal IOP; hence, the development of neuroprotective glaucoma treatment has become a hot research topic at home and abroad (50). In addition, there are systemic therapeutics that can rebalance the pressure gradient of the ethmoid plate by modulating the CSF pressure in patients with POAG who have low CSF pressure and patients with normal intraocular glaucoma. For patients with advanced glaucoma, since most retinal ganglion cells have been apoptotic, stem cell replacement therapy has become the most valuable potential means and needs to become a new hotspot in the treatment of POAG (51).

## Limitations

This bibliometric analysis study inevitably has some limitations. First, it is regrettable that our analysis of global POAG research is somewhat flawed due to the fact that the majority of published articles are in English and there are a large number of journals in other languages that are also worth studying. Second, there are intrinsic differences between the results of bibliometric analysis and real-world studies. For instance, some comparatively new publications of high quality may not attach sufficient attention due to lower citation frequency, whilst older articles have a tendency to accumulate more citations. Thirdly, since the version we used was based on a customized subset (SCI-Expanded, 1999-present) of the whole WoS Core Collection, some excellent articles that are not included in this subset are ignored in this process, which makes the articles lose some luster. Since different databases have different properties including citation frequency counting, document type marking, and export formats for documents, merging of the databases may not optimal choice (52).

## Conclusions

A total of 6,401 articles on POAG research published between 2000 and 2021 were retrieved from the WoSCC SCI-Expanded database. The number of publications, key institutions and countries, published journals, primary authors, and cooperative networks were systematically analyzed using hybrid analysis and visualization technologies (CiteSpace and VOSviewer). The analysis of co-occurrence networks provides researchers with information about potential collaboration opportunities with other institutions and researchers. Bibliometric analyses also reveal the current research hotspots and research frontiers in an objective and comprehensive manner, thus indicating the retrospective view of POAG and providing valuable guidance for researchers in the selection of research topics.

## Author Contributions

Conception and design: LZ, WS, and CW. Data analysis: LZ and JL. Writing: LZ, LF, and CZ. Data collection: WZ, YH, and DW. All authors contributed to the article and approved the submitted version.

## Funding

The study was supported by National Nature Science Fund of China (81974132), National Nature Science Fund of China (81770927), National Key R&D Program of China (2021YFA1101202), Hunan Provincial Health Commission (20220702839).

## Conflict of Interest

The authors declare that the research was conducted in the absence of any commercial or financial relationships that could be construed as a potential conflict of interest.

## Publisher's Note

All claims expressed in this article are solely those of the authors and do not necessarily represent those of their affiliated organizations, or those of the publisher, the editors and the reviewers. Any product that may be evaluated in this article, or claim that may be made by its manufacturer, is not guaranteed or endorsed by the publisher.

## Footnotes

^1^ https://Images.Webofknowledge.Com/Wokrs533jr18/Help/Wos/Hp_Database.Html
