# Supplementary material for: Depicting Developing Trend and Core Knowledge of Primary Open-Angle Glaucoma: A Bibliometric and Visualized Analysis
Source: Front Med (Lausanne). 2022 Jul 5;9:922527. doi: 10.3389/fmed.2022.922527 (PMC9294470; doi:10.3389/fmed.2022.922527)
Supplement: Supplementary file 1 [file Table_1.pdf]

TableS1: The quantity of POAG research in terms of year

Year	Publications	Centrality	Citation
2000	139	2.172	49
2001	135	2.109	345
2002	158	2.468	853
2003	171	2.671	1380
2004	169	2.640	1925
2005	167	2.609	2272
2006	218	3.406	3134
2007	201	3.140	3941
2008	254	3.968	5409
2009	212	3.312	5252
2010	244	3.812	6516
2011	262	4.093	6760
2012	308	4.812	7521
2013	324	5.062	8161
2014	319	4.984	9110
2015	350	5.468	10276
2016	423	6.608	11695
2017	425	6.640	12784
2018	426	6.655	13016
2019	448	6.999	15279
2020	485	7.577	19106
2021	563	8.796	22919
