# Supplementary material for: Depicting Developing Trend and Core Knowledge of Primary Open-Angle Glaucoma: A Bibliometric and Visualized Analysis
Source: Front Med (Lausanne). 2022 Jul 5;9:922527. doi: 10.3389/fmed.2022.922527 (PMC9294470; doi:10.3389/fmed.2022.922527)
Supplement: Supplementary file 2 [file Table_2.pdf]

TableS2 The top 10 countries in the study of POAG

Rank	Country	Publications	Centrality	TLS
1	USA	1898	31.23	1399
2	China	733	12.06	448
3	UK	593	9.76	694
4	Germany	528	8.69	441
5	Japan	510	8.39	184
6	Italy	350	5.76	266
7	India	299	4.92	193
8	South Korea	260	4.28	126
9	Australia	259	4.26	402
10	Turkey	210	3.46	58
